# Chaos Raman distributed optical fiber sensing

**DOI:** 10.1038/s41377-023-01267-3

**Published:** 2023-08-31

**Authors:** Chenyi Wang, Jian Li, Xinxin Zhou, Zijia Cheng, Lijun Qiao, Xiaohui Xue, Mingjiang Zhang

**Affiliations:** 1 College of Physics, Taiyuan University of Technology, Taiyuan, 030024 Shanxi China; 2 Key Laboratory of Advanced Transducers and Intelligent Control System of Ministry of Education, Taiyuan University of Technology, Taiyuan, 030024 Shanxi China; 3 College of Electrical Information and Optical Engineering, Taiyuan University of Technology, Taiyuan, 030024 Shanxi China; 4 Shanxi-Zheda Institute of Advanced Materials and Chemical Engineering, Taiyuan, 030032 Shanxi China

**Keywords:** Imaging and sensing, Optical physics

## Abstract

The physics principle of pulse flight positioning is the main theoretical bottleneck that restricts the spatial resolution of the existing Raman distributed optical fiber sensing scheme. Owing to the pulse width of tens of nanoseconds, the spatial resolution of the existing Raman distributed optical fiber sensing scheme with kilometer-level sensing distance is limited to the meter level, which seriously restricts the development of the optical time-domain reflection system. In this paper, a chaos laser is proposed in the context of the physical principle of the Raman scattering effect, and a novel theory of chaos Raman distributed optical fiber sensing scheme is presented. The scheme reveals the characteristics of chaos Raman scattering light excited by a chaotic signal on the sensing fiber. Further, the chaos time-domain compression demodulation mechanism between the temperature variation information and chaos correlation peak is demonstrated. Then, the position of the temperature variation signal is precisely located using the delay time of the chaos correlation peak combined with the chaos pulse flight time. Based on this novel optical sensing mechanism, an experiment with 10 cm spatial resolution and 1.4 km sensing distance was conducted, and the spatial resolution was found to be independent of the sensing distance. Within the limit of the existing spatial resolution theory, the spatial resolution of the proposed scheme is 50 times higher than that of the traditional scheme. The scheme also provides a new research direction for optical chaos and optical fiber sensing.

## Abstract (web summary)

The first combination of chaos laser and Raman distributed optical fiber sensing breaks the physical bottleneck of spatial resolution, the optimal spatial resolution of the current kilometer-level sensing distance is achieved.

## Graphical abstract

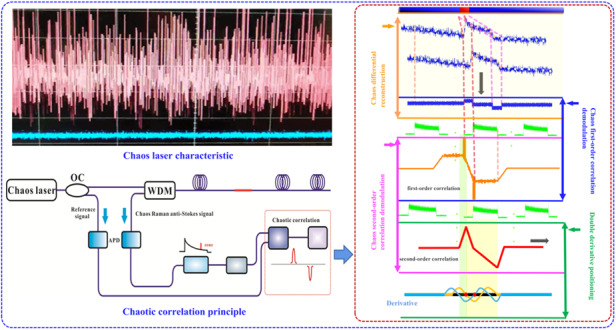

## Introduction

Distributed optical fiber sensing can be applied to the measurement of physical quantities such as strain^1,2^, temperature^3,4^, and vibration^5,6^. Owing to its advantages of long range, immunity to electromagnetic interference, corrosion resistance, and real-time online measurements, it is widely used in many industrial fields.

Spatial resolution represents the smallest spatial unit that can be resolved by the system when detecting the distributed temperature field along an optical fiber^7^. In the traditional Raman distributed optical fiber sensing system, the detection signal is a pulse signal, and its spatial positioning principle is based on the optical time-domain reflection of the pulse-based time-of-flight method^8,9^. Therefore, the Raman anti-Stokes signal generated at a certain position of the sensing fiber is not the light intensity information of the sensing fiber at that position but the superposition of the Raman scattering light intensity information within the length of the sensing fiber corresponding to the entire pulse width^10,11^. This makes it difficult for the system to distinguish the specific location information of the two temperature mutation regions when the distance between them is less than the pulse-width scale^12^. Thus, the spatial resolution of the Raman distributed optical fiber sensing system is limited by the pulse width, which gradually broadens with increase in the sensing distance. This ultimately deteriorates the spatial resolution of the system to several meters or even tens of meters at the end of the optical fiber^12–16^.

Compression of the pulse width of the optical source can further improve the spatial resolution. However, this scheme will also deteriorate the signal-to-noise ratio (SNR) of the system, which affects the performance of the sensing distance. Therefore, the existing Raman distributed optical fiber sensing system has a technical bottleneck in that the SNR and spatial resolution cannot be balanced^17–19^. For example, Ososkov et al. demonstrated an ultrashort pulse-mode-locked fiber laser scheme by compressing the pulse width. The experimental results showed that this scheme achieves a spatial resolution of 10 cm and sensing distance of 3.0 m^20^. Researchers have proposed many advanced solutions to optimize the spatial resolution of the fiber-optics sensing system while ensuring the SNR of the system. It mainly includes a pulse modulation scheme based on a single-mode fiber^20–22^ and few-mode fiber^23,24^. The pulse modulation scheme based on a single-mode fiber can increase the luminous flux coupled to the sensing fiber by suppressing the nonlinear effect of the fiber. Furthermore, the single-mode fiber ensures that the spatial resolution does not deteriorate with increase in the length of the sensing fiber. For instance, Sun et al. proposed and experimentally proved a genetically optimized aperiodic code scheme, and a spatial resolution of 1.0 m was obtained for a sensing distance of 39.0 km^18^. In another scheme, the few-mode fiber has a larger mode-field area and higher nonlinear threshold, which can enable the coupling of a larger incoming optical power to the sensing fiber. Compared with the multimode sensing scheme, the few-mode fiber can achieve quasi-fundamental mode transmission. He et al. developed a graded-index few-mode fiber with a large effective area and low dispersion and achieved a spatial resolution of 1.13 m at a sensing distance of 25.0 km^24^.

However, these advanced schemes cannot overcome the degradation of the spatial resolution to the meter level. In 2021, we proposed a simulation model based on chaotic and Raman scattering signals^25^. In the present study, a chaos Raman distributed optical fiber sensing scheme was experimentally demonstrated. The experimental results showed that the system could achieve a spatial resolution of 10 cm over a fiber length of 1.5 km under a 50 ns pulse width. The proposed scheme improves the spatial resolution of the traditional demodulation scheme (pulse time positioning method) by 50 times. To the best of our knowledge, this is the first time that a spatial resolution of 10 cm has been achieved at a sensing distance in the range of kilometers.

## Results

### Experimental setup

**Fig. 1:**
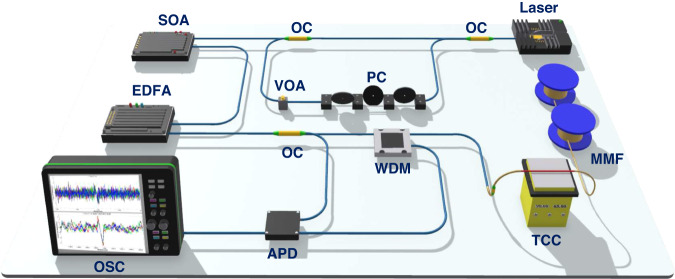
Experimental setup of chaos Raman distributed optical fiber sensing scheme

We propose a novel scheme to examine the sensing distance and spatial resolution of a chaotic Raman distributed optical fiber sensing system using chaos differential reconstruction and the chaos double correlation method. A chaos laser was used as the light source, and its autocorrelation characteristics were used to eliminate the influence of the chaos pulse width of the light source on the spatial resolution of the system. The weak detection signals were enhanced through double correlation algorithms and derivative analysis. The experimental setup is illustrated in Fig. 1. After the traditional laser with a center wavelength of 1550 nm passes through the circulator, it is modulated into continuous chaotic light through a single feedback structure comprising an attenuator, a polarization controller, and fiber coupler. The continuous chaos light is modulated by a semiconductor optical amplifier (SOA) into chaos pulse light and then amplified by an erbium-doped fiber amplifier (EDFA). Then, the chaos pulse is divided into two parts by a 1:99 coupler, where 1% is used as the chaos pulsed reference signal of the correlation algorithm, and 99% enters the sensing fiber through the wavelength division multiplexer (WDM) to generate the Raman backscattered signal. The WDM can filter anti-Stokes light with a wavelength of 1450 nm. The anti-Stokes signal and chaos pulsed signal are converted into electrical signals by an avalanche photo diode (APD) and collected by an oscilloscope (OSC).

### Positioning results

**Fig. 2:**
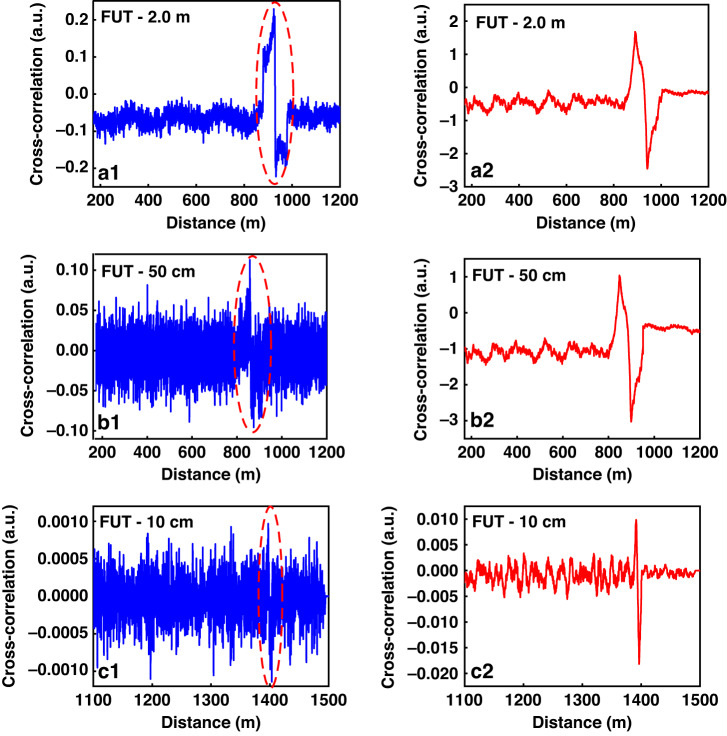
Positioning results of chaos Raman distributed optical fiber sensing scheme. **a**1 Demodulation result by using chaos first-order correlation demodulation for a 2.0 m FUT, **a**2 demodulation result by using chaos second-order correlation demodulation for a 2.0 m FUT, **b**1 demodulation result by using chaos first-order correlation demodulation for a 50 cm FUT, **b**2 demodulation result by using chaos second-order correlation demodulation for a 50 cm FUT, **c**1 demodulation result by using chaos first-order correlation demodulation for a 10 cm FUT, **c**2 demodulation result by using chaos second-order correlation demodulation for a 10 cm FUT

Figure 2 shows a comparison of the results of the chaos first-order and second-order correlations. Figure 2a1–c1 shows the corresponding experimental results after chaos first-order correlation under the condition that the optical fiber sensing distance is 1.0–1.5 km. The chaos pulse widths for FUTs (Fiber Under Text) of length 2.0 m, 50 cm, and 10 cm were 500.0 ns, 500.0 ns, and 50.0 ns, respectively. As the chaos pulse width increases, the corresponding noise increases; therefore, a 50.0 ns chaos pulse was selected for an FUT of length 10 cm to reduce the influence of noise on the demodulation results. The corresponding chaos second-order correlation demodulation results are shown in Fig. 2a2–c2.

In the traditional demodulation scheme, the system can achieve a spatial resolution of only 5.0 m owing to the limitation of the pulse width. In the chaotic Raman distributed optical fiber sensing system, the temperature variation region can be identified even when it is much smaller than the pulse width, which proves that the spatial resolution of the system can overcome the limitation of the pulse width. As can be seen from Fig. 2a1, when the length of the FUT is in the order of meters, the correlation peak is relatively obvious, but as can be seen from Fig. 2b1–c1, when the FUT zone is shorter than one meter, only the change trend of the positive and negative peaks can be seen, but it is difficult to precisely locate the position and value of the peak, which will affect the temperature accuracy of the system.

The demodulation results obtained after performing chaos second-order correlation processing on the signal are shown in Fig. 2a2–c2. It can be clearly observed that this scheme can effectively enhance useful signals and reduce noise, particularly when the length of the FUT is less than 1.0 m, as shown in Fig. 2b2, c2.

**Fig. 3:**
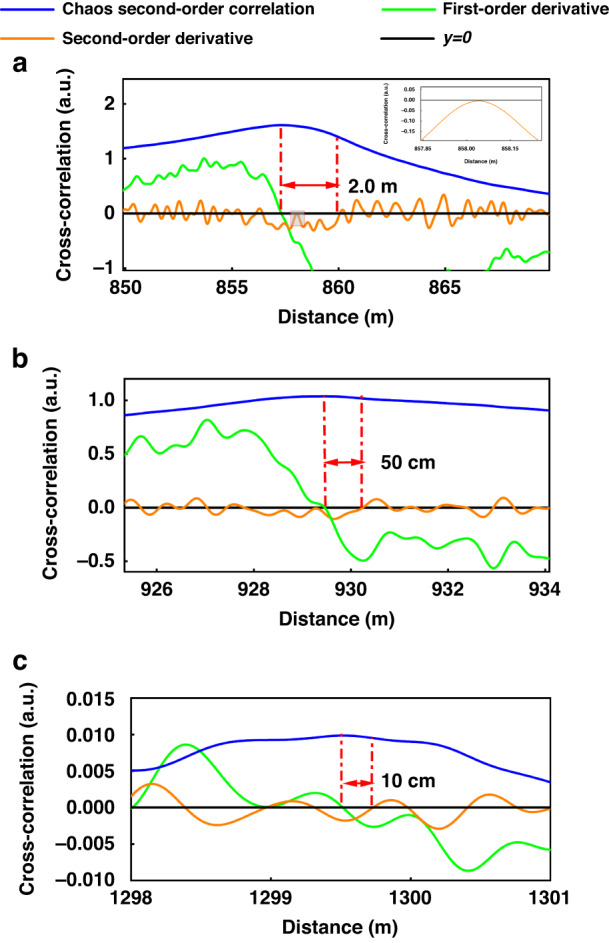
Positioning results. **a** FUT length 2.0 m, **b** FUT length 50 cm, **c** FUT length 10 cm (green and orange curves are used to indicate the starting and ending positions of the FUT)

After obtaining the obvious peak-to-peak correlation value, the corresponding temperature can be obtained using the demodulation equation. Figure 3 shows the positioning results of the FUT zone corresponding to the system under the chaotic second-order correlation demodulation. The blue curve is the correlation peak curve after the chaos second-order correlation, and the green and orange parts are the first- and second-order derivatives of the chaos correlation curve, respectively. The black line is the axis of *y* = *0*, which is convenient for locating the point where the derivative is zero. We use the point with a zero first-order derivative to locate the starting position of the FUT zone, after which the nearest point where the second-order derivative is zero is used to locate the end of the FUT zone, which, in turn, can precisely determine the length of the FUT zone.

In Fig. 3a, the starting position of the FUT is located first. The end point of the FUT is expected to occur behind this position. After enlarging, we can see that this point does not intersect the axis of *y* = *0*; hence, it can be ignored. The first point at which the second-order derivative is zero is the end of the FUT. The distance between the starting and ending points is 2.0 m, which is consistent with the actual experimental setup. Similarly, Fig. 3b, c corresponds to the localization results when the FUT is 50 cm and 10 cm, respectively. Through the above method, clear and accurate localization can be achieved, and the spatial resolution of the system can be improved.

### Temperature demodulation results

We proposed Raman distributed optical fiber sensing based on the chaos double correlation method. It uses the positive correlation peak–peak coefficient of the time-domain compression and demodulation method of the correlation function to extract the abrupt temperature change along the sensing fiber.

To verify the feasibility of the scheme, we placed fiber rings with lengths of 2.0 m, 50 cm, and 10 cm at the end of the fiber. The temperature was set to 60.00 °C, 70.00 °C, 80.00 °C, and 90.00 °C using the temperature control platform (TCP). The rest of the fiber was placed in the normal temperature area at approximately 25.00 °C. Then, chaotic pulse signals of 500 ns were injected into the sensing fiber with FUT of 2.0 m and 50 cm, respectively. Chaotic pulse signal of 50 ns was injected into the sensing fiber with FUT of 10 cm, and the correlation function was compressed and demodulated in the time domain based on the reconstructed Raman anti-Stokes scattering signal and chaos reference signal under the conditions of this model.

**Fig. 4:**
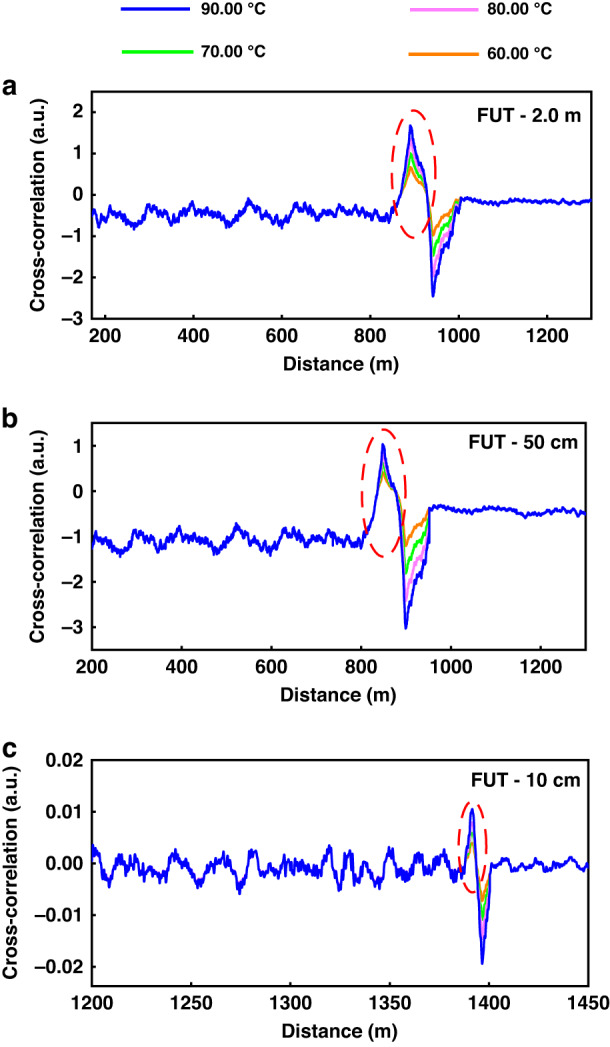
Relationship between chaos correlation peaks and temperature for **a** 2.0 m FUT, **b** 50 cm FUT, and **c** 10 cm FUT

Chaos Raman backscattered signals and chaotic pulse signals were acquired by the OSC and used for subsequent chaos differential reconstruction and chaos double correlation processing. The blue, pink, green, and orange curves in Fig. 4 correspond to temperatures of 90.00 °C, 80.00 °C, 70.00 °C, and 60.00 °C, respectively.

**Fig. 5:**
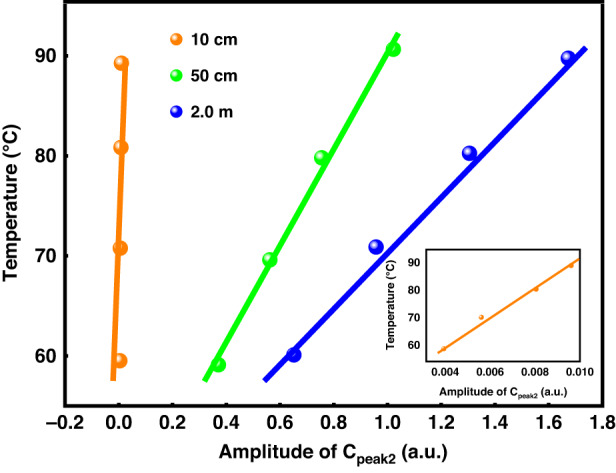
Temperature demodulation results for various FUT lengths

To obtain specific temperature data corresponding to the correlation peak, we can demodulate the corresponding temperature using Eq. (10) (The derivation process of Eq. (10) is detailed in section 5. Materials and methods: D. Physical characteristics of chaos correlation peaks and surrounding temperature) the results are presented in Fig. 5. The data were fitted to obtain blue, green, and orange lines corresponding to FUT zone lengths of 2.0 m, 50 cm, and 10 cm, respectively.

${\sum }_{j=0}^{m-1}[{P}_{r}(j){P}_{r}(j+1)-{P}_{r}^{2}(j)]$ is related only to the width of the chaotic pulse signal. When the pulse width is fixed, this term is fixed; therefore, the temperature demodulation result *T*_*FUT*_ is determined only by *A* and is positively correlated, where *A* is related only to the correlation *C*_*peak2*_. Therefore, the results of the temperature demodulation are affected only by the correlation peak–peak values, which are directly proportional to each other. It can be seen from Fig. 5 that the temperature demodulation results and correlation peak–peak values are linearly correlated; that is, the experimental results are in good agreement with the theoretical analysis results, which proves the feasibility of the quadratic correlation method. The double correlation method can accurately identify the temperature change and obtain accurate temperature information.

In terms of long-distance sensing, because the proposed chaotic correlation scheme is to demodulate the temperature variation information along the sensing fiber through the correlation processing of chaotic reference signal and chaotic Raman signal after differential reconstruction to obtain the correlation peak. The distortion of chaotic signal caused by optical fiber dispersion can affect the correlation characteristics of the two signals, that is, the peak coefficient of chaos correlation peak will be affected, but the position of chaos correlation peak will not be affected. We demodulated the position of FUT based on the position of correlation peak and calculated the temperature of FUT based on correlation peak–peak coefficient. Therefore, the fiber dispersion could affect the temperature accuracy or SNR performance of chaos Raman distributed optical fiber sensing scheme but will not affect the sensing spatial resolution.

## Discussion

### Temperature accuracy

In chaos Raman distributed optical fiber sensing, the second-order correlation scheme and analysis of the double-derivative demodulation method can optimize the measurement temperature sensitivity of the system. The chaos correlation peak generated by this scheme is formed by the compression of the Raman anti-Stokes scattering signal carrying the temperature change information in the length range of the FUT to the initial position of the FUT in the time domain so that the multiplier Raman temperature effect can be generated at this position. In addition, the reconstructed Raman anti-Stokes signal based on chaos differential reconstruction technology can reduce the influence of the non-temperature mutation regions on the reconstructed signal and further enhance the SNR of the Raman anti-Stokes scattering signal modulated by the ambient temperature in the FUT region. Based on the anti-interference characteristics of the correlation function, correlation compression, and demodulation technology, the system can achieve good temperature sensitivity on a small scale. The temperature measurement analysis is shown in the Table 1. The parameters of superimposed Raman scattering signal is shown in the Table 2. As can be seen, the temperature accuracy can be controlled within 1.32%.

**Table 1:** Temperature measurement analysis

FUT	Temperature of TCC	Measurement temperature	Measurement error
2.0 m	90.00 °C	89.76 °C	0.27%
	80.00 °C	80.26 °C	0.32%
	70.00 °C	70.89 °C	1.27%
	60.00 °C	60.11 °C	0.18%
50 cm	90.00 °C	90.66 °C	0.73%
	80.00 °C	79.82 °C	0.23%
	70.00 °C	69.62 °C	0.54%
	60.00 °C	59.21 °C	1.32%
10 cm	90.00 °C	89.27 °C	0.81%
	80.00 °C	80.84 °C	1.05%
	70.00 °C	70.78 °C	1.11%
	60.00 °C	59.52 °C	0.80%

**Table 2:** Parameters of superimposed Raman scattering signal

Parameters	Symbol
Incident power of chaos pulsed laser	*P*
Raman anti-Stokes backscattering coefficient	*K*
Backscattered factor of optical fiber	*S*
Wavelength of anti-Stokes scattering signal	*λ*_*as*_
Luminous flux	*ϕ*_*e*_
Loss coefficient	*α*_*0*_ + *α*_*as*_
Length of sensing optical fiber	*L*
Temperature modulation function	*R*_*as*_(*T*)

### Sensing distance and SNR

**Fig. 6:**
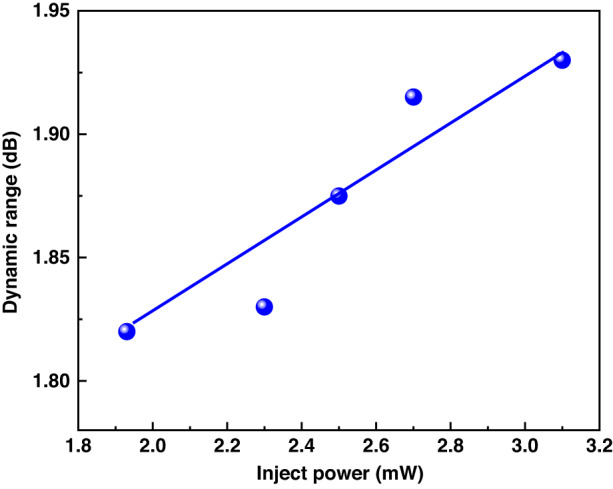
The relationship between the incident power and the dynamic range of Raman backscattering signal

The sensing distance of the system is directly determined by the incident chaotic light power. So we first study the influence of chaotic pulse incident power on the sensing distance of the system. In the experiment, we control the pulse incident power by adjusting the bias current of the EDFA. In the Raman backscattering signal based on the principle of optical time domain reflection positioning, the dynamic range of the backscattering signal is directly related to the sensing distance. Therefore, we studied the relationship between the dynamic range of the system and the incident power in the experiment. The relationship between the incident power and the dynamic range of Raman backscattering signal is shown in Fig. 6. The experimental results show that the dynamic range of the system is gradually increasing with the increase of the incident power, which means that the sensing distance of the system will gradually increase. However, due to the influence of the maximum tolerable optical power of the device and the stimulated Raman scattering threshold, the incident power of the system cannot continue to increase. For example, the output power of EDFA is too large, the wavelength division multiplexer (WDM) will be damaged. Therefore, in the case of better instruments, we can achieve distributed temperature sensing with higher dynamic range and longer sensing distance.

In addition, the incident power is also directly related to the signal-to-noise ratio (SNR) of the system. The chaotic correlation scheme proposed in this paper is to use the chaotic correlation peak to locate the temperature change signal along the sensing fiber. Therefore, we added a experiment on the positioning and demodulation of chaotic correlation peaks in the FUT region based on different incident optical powers.

**Fig. 7:**
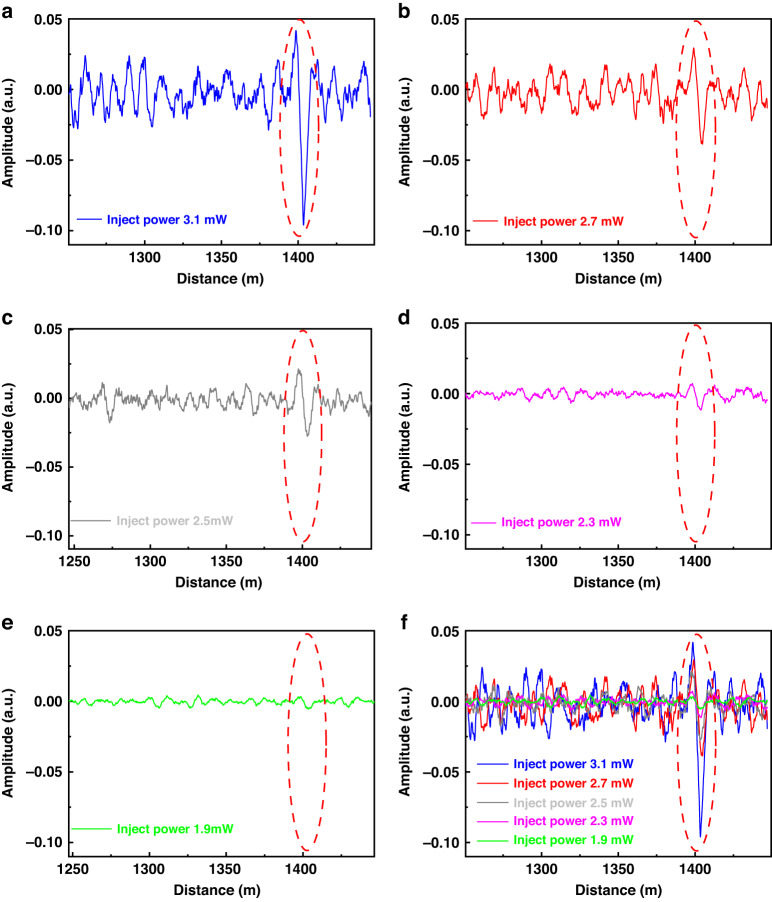
Chaotic correlation demodulation signals under different inject optical power. **a** 3.1 mW, **b** 2.7 mW, **c** 2.5 mW, **d** 2.3 mW, **e** 1.9 mW, **f** demodulation signals under the different inject power

The experimental results are shown in Fig. 7. As the incident power gradually increases, the chaotic correlation peaks in the FUT region along the sensing fiber are also easier to distinguish. Therefore, under the premise that the device is not damaged and the signal is not stimulated. the larger the incident optical power, the stronger the SNR of system.

### Spatial resolution

Then we theoretically and experimentally study and analyze the influence of pulse width, chaotic sub-pulse number and chaotic time delay signature (TDS) on the sensing spatial resolution. After theoretical and experimental analysis, it is concluded that chaotic signals under different modulation pulse widths do not affect the sensing spatial resolution. However, due to the influence of the TDS, the SNR performance of the sensing system will be deteriorated when the modulation pulse width is too large, resulting in the FUT region along the sensing fiber cannot be effectively identified. The theoretical analysis and experimental results are as follows.

**Fig. 8:**
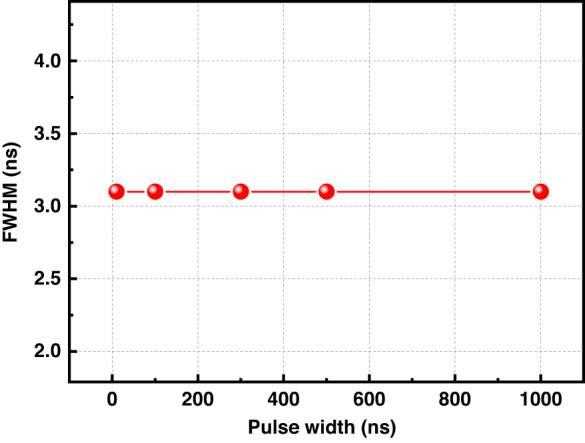
Relationship between the different pulse widths and FWHM of ACF of chaotic signals

The sensing spatial resolution of the chaotic correlation demodulation scheme proposed in this paper depends on the full width at half maximum (FWHM) of the autocorrelation function of the chaotic signal. We carried out the FWHM measurement experiment of chaotic signal autocorrelation function (ACF) under different pulse width control. The experimental results are shown in Fig. 8. The FWHM of ACFs with different pulse widths are 3.1 ns, which means that the pulse width of chaotic signals does not affect the FWHM of ACF of chaotic signals. This also proves that the advantage of the chaotic correlation demodulation scheme proposed in this manuscript is that it can break the technical bottleneck that the spatial resolution of traditional system is limited by the pulse width.

**Fig. 9:**
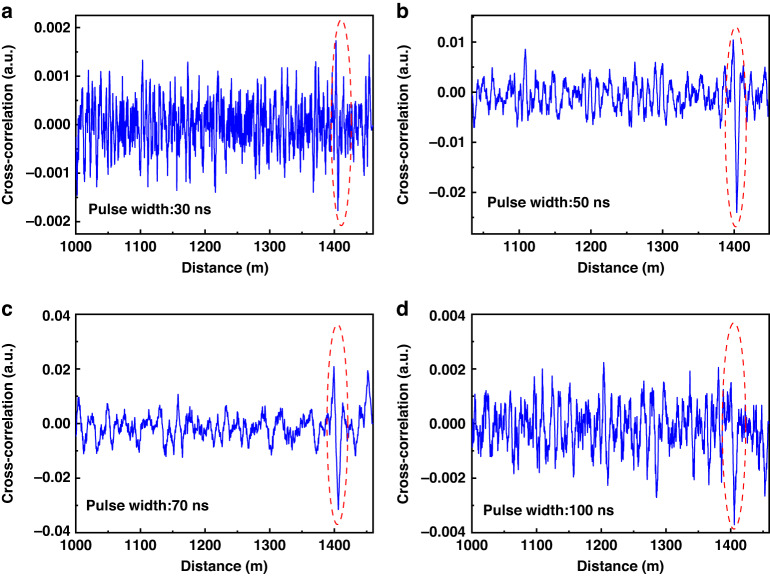
Chaotic correlation demodulation positioning results under different pulse widths. **a** Pulse width 30 ns, **b** pulse width 50 ns; **c** pulse width 70 ns; **d** pulse width 100 ns

In addition, the pulse width of the chaotic signal will affect the number of chaotic sub-pulses, which will affect the system SNR and ultimately affect the sensing performance. Therefore, we added a relevant experiment. In the experiment, we modulate the chaotic pulse signals with four pulse widths of 30 ns, 50 ns, 70 ns and 100 ns by a pulse modulator, and then perform chaotic correlation demodulation on the FUT region along the sensing fiber under the other same experimental conditions. The experimental results are shown in Fig. 9. The results show that the chaotic signal with modulation pulse width of 50 ns and 70 ns as the detection signal can accurately locate the FUT region. However, the correlation peak of the chaotic signal with 30 ns pulse width has been submerged in the ambient temperature signal, and the position of the FUT region cannot be effectively identified along the sensing fiber. This is because the larger the pulse width of the chaotic signal, the more chaotic sub-pulses it contains. This phenomenon causes the stronger the correlation signal between the chaotic Raman backscattering signal after differential reconstruction and chaotic reference signal, resulting in a stronger SNR of the sensing system. Therefore, in the experiment, when the modulation pulse width is 30 ns, the random fluctuation characteristic contained in the chaotic Raman backscattering signal reconstructed by differential reconstruction method is less, resulting in the excited chaotic correlation peak being submerged in the surrounding temperature measurement signal, which cannot effectively identify the FUT region along the sensing fiber.

**Fig. 10:**
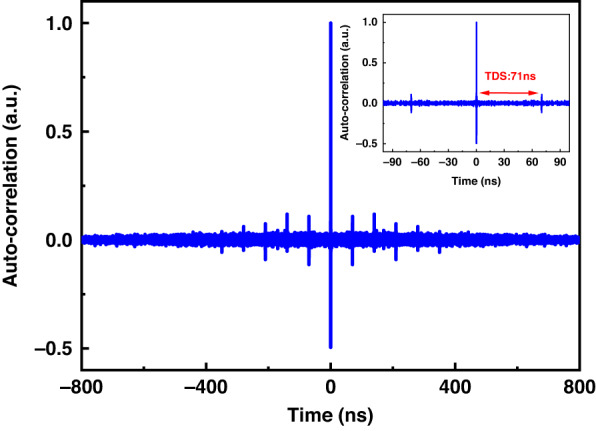
The autocorrelation function curve of chaotic signal when the pulse width length is greater than the TDS of chaos signal

However, the chaotic signals with longer pulse width will lead to the generation of TDS. The reason is that for the single feedback external cavity chaotic laser source with fixed feedback cavity length, the chaotic laser generated by the delayed optical feedback structure has a certain periodicity, which is characterized by a series of weak amplitude autocorrelations on both sides of the chaotic autocorrelation peak, namely TDS. As shown in Fig. 10, there are a series of secondary correlation peaks (also known as the value of side lobe in ACF) on both sides of the chaotic autocorrelation center peak. In the experiment, we study the TDS information of the single feedback chaotic signal device, and the TDS is 71 ns. In the chaotic correlation demodulation scheme proposed in this paper, if the modulation pulse width length is greater than the TDS of the chaotic signal, each order noise peak will be excited in the sensing fiber. At the same time, the base signal of the ACF curve also excites the weak noise field that accumulates with the length of the sensing fiber. Therefore, the signal carrying the temperature change information in the chaotic correlation peak is easily overwhelmed by various orders of noise, which ultimately leads to the FUT position cannot be effectively identified, and the sensing performance is greatly reduced (as shown in Fig. 9d).

**Fig. 11:**
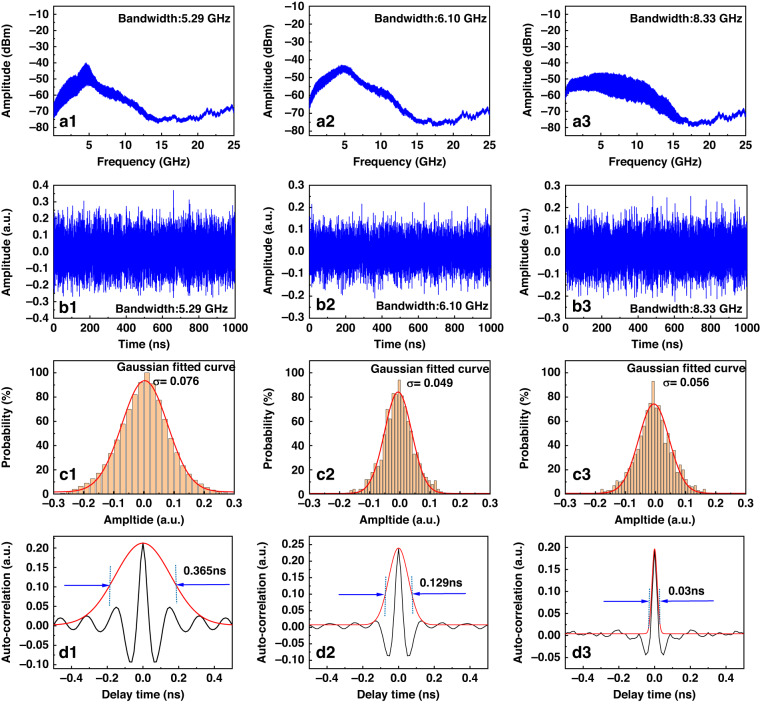
Chaotic state characteristics under different chaotic bandwidths. Spectrum at bandwidth (**a**1) 5.29 GHz, (**a**2) 6.10 GHz, (**a**3) 8.33 GHz. Timing at bandwidth (**b**1) 5.29 GHz, (**b**2) 6.10 GHz, (**b**3) 8.33 GHz. Probability density distribution of the amplitude of sub-pulses at bandwidth (**c**1) 5.29 GHz, (**c**2) 6.10 GHz, (**c**3) 8.33 GHz. The FWHM at bandwidth (**d**1) 5.29 GHz, (**d**2) 6.10 GHz, (**d**3) 8.33 GHz

In addition, we theoretically and experimentally study the influence of the bandwidth, probability density distribution of the amplitude of sub-pulses and spectral shape of chaotic signals on the sensing spatial resolution. The probability density distribution of the amplitude of sub-pulses and spectral shape are mainly affected by the bandwidth of chaotic signals. Therefore, in the experiment, we control the feedback light intensity of the injected laser by adjusting the attenuator and polarizer in the feedback loop of the chaotic laser generation device and obtain three groups of chaotic lasers with different states (bandwidth). as shown in the Fig. 11a1–a3 shows the spectrum corresponding to different chaotic states, and their standard bandwidths (80%) are 5.29 GHz, 6.10 GHz and 8.33 GHz, respectively. Figure 11b1–b3 shows the chaotic time series. The Fig. 11c1–c3 is shown as the corresponding time series amplitude probability distribution, and the amplitude of the chaotic signal satisfies the Gaussian probability distribution. Figure 11d1–d3 shows the FWHM corresponding to different bandwidths.

The sensing spatial resolution of chaotic Raman distributed optical fiber sensing scheme is related to the FWHM of ACF. As shown in Fig. 11d1–d3, the larger the bandwidth of the chaotic signal, the narrower the FWHM of the ACF, which means that the sensing spatial resolution performance is better.

However, in practical experiments, due to the Raman backscattering signal is only the order of nanowatts, the bandwidth of the existing commercial APD for Raman backscattering signal acquisition is only several hundred MHz. Therefore, the performance of the APD device in the actual experiment also limits the further improvement of the sensing spatial resolution of this proposed scheme. If the commercial APD bandwidth based on Raman distributed optical fiber sensing system is further improved in the future, the sensing spatial resolution using the chaotic correlation demodulation can be expected to break through to millimeter-level or even submillimeter-level.

### Compared with the pulse coding scheme

Compared with the conventional pulse coding scheme^18,19,26,27^, the chaotic correlation scheme proposed in this paper has the advantage that this scheme can break the limitation of the pulse width of the laser source on the sensing spatial resolution performance. The localization principle of chaotic correlation scheme is the correlation positioning, and its sensing spatial resolution is determined by the full width at half maximum (FWHM) of chaotic autocorrelation function.

The conventional pulse coding scheme (e.g., analog modulation with constant amplitude) is based on the positioning principle of OTDR, and its spatial resolution is also limited by the single bit pulse width of the laser source, which is consistent with the traditional Raman distributed optical fiber sensing system. The pulse coding scheme can suppress the laser source noise, so as to improve the light flux coupled to the sensing fiber. Then the spatial resolution is improved by reducing the pulse width of the laser source, so as to improve both the SNR and the spatial resolution performance of the sensor. For example, in the reference^19^, Taki et al. use the cyclic pulse coding scheme based on the 10 ns single bit pulse width realized the spatial resolution of 1.0 m on the sensing distance of 10.0 km; In the reference^28^, Park et al. use the cyclic pulse coding scheme based on the 100 ns single bit pulse width realized the spatial resolution of 17.0 m on the sensing distance of 37.0 km. However, due to the limitation of the pulse width, its spatial resolution is still not better than 1.0 m. Fortunately, we achieved a spatial resolution of 0.1 m based on a chaotic pulse laser source of 50 ns in this paper. The traditional Raman distributed optical fiber sensing demodulation system and pulse coding scheme can only achieve a sensing spatial resolution of 5.0 m at this pulse width.

The amplitude of each sub-pulse in the above conventional pulse coding signal is constant, while the amplitude of each sub-pulse signal in the chaos pulse signal is random. The sensing spatial resolution of the conventional Raman distributed optical fiber sensing system is limited by the pulse width because the signal collected by the system at each position point is the superposition of all scattered signals within the length corresponding to the pulse width of the laser. The proposed differential reconstruction method is designed to extract the Raman scattering signal information compressed at each position point within the length of the pulse width. This scheme is also based on the amplitude randomization of each sub-pulse of the chaos signal to achieve the differential reconstruction of the Raman anti-Stokes scattering signal, and then correlate the demodulation with the original chaos signal of the reference path to precisely locate the FUT position information. In contrast, the conventional coded pulse signals with constant amplitude modulation (e.g., Gray codes, Simple codes, etc.) cannot use the proposed differential reconstruction scheme to reconstruct the Raman scattered signals due to the constant signal amplitude of their sub-pulses, and thus cannot use the correlation method to locate the position information and demodulate the temperature information of the FUT.

In addition, some special pulse coding scheme with non-constant amplitude modulation (e.g., analog modulation with random amplitude) can realize the random time series strength. Generally, there are three modulation schemes that can realize the random amplitude characteristics. The detailed modifications are as follows.

Firstly, the optical signals with random amplitude can be generated by modulating laser diode or the EOM (Electro-optic Modulators) utilizing electric noise signal. Our group used this scheme to generate random amplitude laser signals in previous work^29^. In this paper, the optical chaotic signal is directly generated based on the optical feedback configuration. Compared with the above-mentioned signal with electrical noise modulation, the chaotic signal has the characteristics of wider bandwidth. In this paper, the bandwidth of optical chaotic signal can reach up to 8.33 GHz. However, the bandwidth of the signal modulated by electrical noise can only reach the dozens of MHz to 1.4 GHz^29,30^. This phenomenon means that the proposed chaotic correlation scheme can achieve a higher sensing spatial resolution in theory. This is because in the correlation demodulation scheme, the sensing spatial resolution is related to the FWHM of the autocorrelation function of the sensing signal. The larger the bandwidth, the narrower the FWHM, and the higher the sensing spatial resolution. Furthermore, the direct generation of chaotic light in optical domain is relatively simple and inexpensive, such as the chaotic signals can be generated only by laser diode with single-feedback configuration including attenuators, optical coupler, and polarization controller.

Secondly, the optical signals with random amplitude also can be generated by modulating laser diode utilizing the electric chaotic signal. The group in our laboratory also used electric chaotic modulation to generate random amplitude signals in previous work^31–33^. In the experiment, the bandwidth generated by electric chaotic signal can only reach 1.7–1.8 GHz^32,33^. In addition, the definition of the bandwidth of the electrical chaotic signal in references^32,33^ are the definition of full bandwidth. If a 3 dB bandwidth definition is used, the bandwidth of the above electrical chaotic signal will be smaller. Therefore, the sensing signal generated by electric chaotic signal modulation will also limit the sensing spatial resolution of the system.

Thirdly, some light sources can directly output the optical laser signals with random amplitude characteristics, such as ASE (amplifier spontaneous emission) signal. Compared with the chaotic laser, its unit power spectral density is relatively lower. This means that the signal-to-noise ratio (SNR) of the Raman scattering signal is relatively low, which ultimately affects the sensing distance of the system. In our previous work, we have verified the theoretical possibility of the correlation demodulation method based on the ASE signal^14^. However, when the ASE signal was used as the sensing signal in experiment, we found that the sensing distance can only be achieved by tens of meters. In addition, the spectrum of ASE signal is wider than that of chaotic signal, which means that the dispersion effect of ASE signal in multimode fiber sensing is relatively larger. In the detection of long sensing distance, the larger dispersion will affect the correlation performance between the reference signal and differential reconstruction Raman scattering signal, and ultimately affect the SNR of the system.

However, the proposed chaotic correlation demodulation scheme also has some drawback, if we want to obtain higher spatial resolution, it requires the bandwidth of the detector of the system to be large. And the sampling rate of the acquisition system is required to be high, so that the high spatial resolution can be realized.

### Anti-interference characteristics of chaotic sensing signal

In this paper, we not only replace the chaotic sensing signal with the traditional pulse signal, but also replace the traditional OTDR positioning principle with the correlation positioning principle. The one of the advantages of chaotic signal as a sensing signal is its strong anti-interference ability. This is because in the proposed chaotic correlation scheme, the chaotic reference signal can only produce a strong correlation peak with chaotic sensing signal whose amplitude characteristics are almost the same to its own, and will not be related with other noise signals. In the chaotic Raman distributed fiber sensing system, after the signals scattered by the sensing fiber are filtered by a WDM (wavelength division multiplexer, the filtered wavelength is 1450 nm), only the chaotic Raman scattering signal after differential reconstruction process can be correlated with the chaotic reference signal. Compared with the other types of sensing signals, the chaotic signal combined with the correlation demodulation scheme has a stronger anti-interference ability, which can suppress some noise components and improve the SNR of the system. In addition, the anti-interference advantage of chaotic signals combined with the correlation scheme, has been demonstrated in the field of radar detection^34,35^.

Although some chaos sub-pulses signal with weaker energy will increase the base noise of the system. However, based on the anti-interference characteristics of chaotic sensing signal, the whole sub-pulses of the chaos laser play an important role in chaos differential reconstruction and correlation demodulation process. And each sub-pulse has improved the correlation performance of the system. This is because all the sub-pulse sequences will maintain the time-series random fluctuation characteristics of the chaos signal in the excited Raman scattering signal when passing through the FUT region.

In conclusion, a chaotic Raman distributed optical fiber sensing scheme was experimentally proposed. The scheme reveals the characteristics of chaotic Raman scattering light excited by chaotic signals on the sensing fiber. The time-domain compression modulation mechanism between the distributed temperature information and chaos correlation peak was demonstrated. Then, the position of the temperature variation signal was precisely located based on the delay time of the chaos correlation peak combined with the pulse flight time. The demodulation results show that the novel optical sensing mechanism allows the system to overcome the limitation of the pulse width in terms of the spatial resolution. For a chaos pulse width of 50.0 ns, the proposed scheme can achieve 10 cm spatial resolution without sacrificing the sensing distance. Within the limits of the existing spatial resolution theory, the spatial resolution of the chaotic Raman distributed optical fiber sensing scheme is 50 times higher than that of the traditional scheme. To the best of our knowledge, this is the first time that a high spatial resolution of 10 cm has been achieved over an optical fiber sensing distance in the order of kilometers. In the future, the chaos Raman distributed optical fiber sensing scheme will continue to be studied in terms of long sensing distance.

## Materials and methods

### Chaos characteristics

Chaos laser is a new type of laser with the characteristics of a noise-like and wide spectrum. Its random amplitude characteristics in the time series can be used to obtain the chaotic Raman backscattered signal of a single point along the sensing fiber, which offers great advantages in improving the spatial resolution of long-distance distributed optical fiber sensing systems. The chaos differential reconstruction algorithm can separate chaotic random fluctuation characteristics, and the correlation algorithm uses the random fluctuation characteristics to obtain the temperature information.

**Fig. 12:**
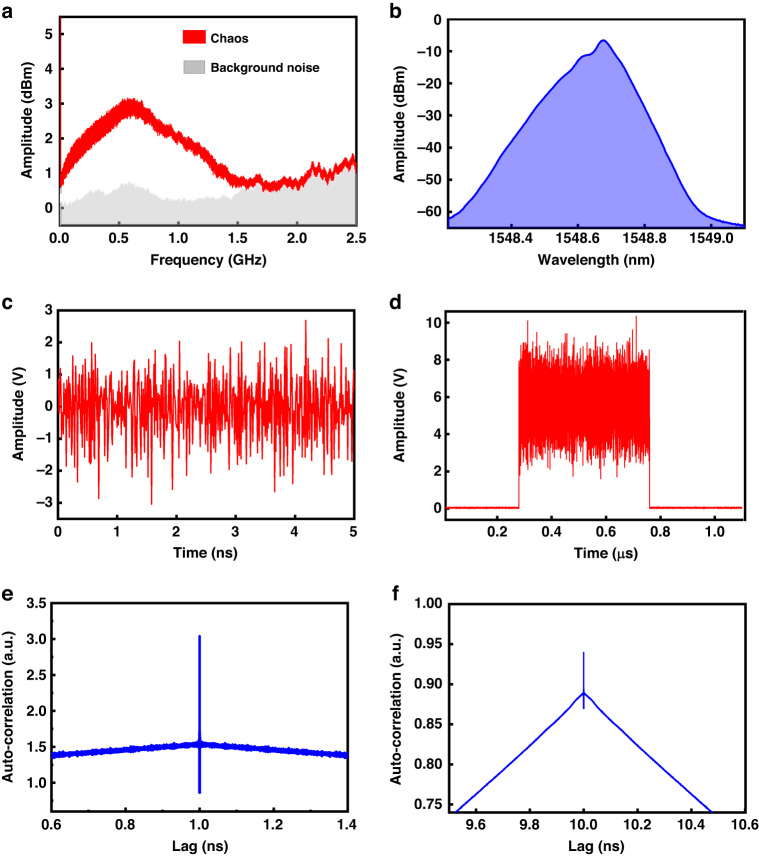
Chaos signal characteristics, (**a**) power spectra, (**b**) optical spectrum, (**c**) continuous time series, (**d**) chaos pulse time series, (**e**) autocorrelation characteristic of chaos signal, (**f**) autocorrelation characteristic of chaotic pulse signal

Figure 12 shows the analysis of the chaos characteristics. Figure 12a shows the chaos power spectra, which are wider than those of traditional light. Figure 12b shows the optical spectrum of the chaos laser. In comparison with the conventional laser, the chaos laser shows wider spectral characteristics and lower coherence. Figure 12c, d shows the timing diagrams of the continuous chaos light and pulsed chaos light, respectively. The chaotic laser exhibits random amplitude characteristics, which can be used to identify the regions of minimal temperature variations in the sensing fibers. Figure 12e, f shows the autocorrelation characteristics of chaos continuous light and chaos pulsed light, respectively. It can be seen that the chaos laser has a strong anti-interference ability and characteristics similar to those of the delta function.

### Chaos pulse transmission and superposition characteristics of chaos Raman scattering signal

Raman optical fiber sensing is based on the principle of Raman scattering, which is a type of optical scattering where the interaction of a pulsed light with molecular motion changes the frequency of the incoming light when it passes through the sensing fiber. The pulsed light either absorbs or emits optical phonons from or to the sensing fiber, subsequently converting it into an anti-Stokes light with a high frequency. The anti-Stokes signal is expressed by Eq. (1):

1 $${I}_{a}(L)=P\cdot K\cdot S\cdot {\phi }_{e}\cdot {R}_{as}(T)\cdot {{\lambda }_{as}}^{-4}\cdot \exp [-({\alpha }_{0}+{\alpha }_{as})L]$$

2 $${R}_{as}(T)={\left[\exp \left(\frac{h\varDelta v}{kT}\right)-1 \right]}^{-1}$$

where Δ*ν* is the Raman frequency shift, *h* is Planck’s constant, *k* is Boltzmann’s constant, and *T* is the temperature of the sensing fiber.

**Fig. 13:**
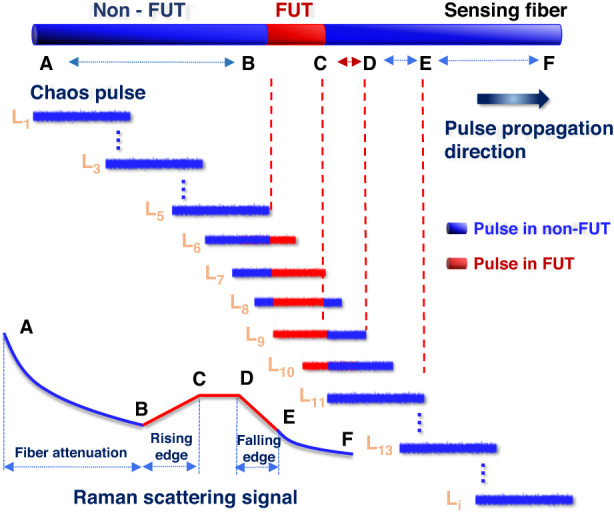
Superposition characteristics of chaos Raman backscattered signals in the fiber under test (FUT) region

Figure 13 shows the generation process of chaotic Raman scattering signals when the pulse width is significantly larger than the length of the fiber under test (FUT) zone. The A-B segment is the stage in which the pulse has not yet entered the FUT zone. Owing to the attenuation of light, the scattering signal exhibits a uniform downward trend.

B-C segment and D-E segment are the phases during which the pulse enters and leaves the FUT zone, respectively; these correspond to the rising and falling edges in the graph shown in the figure. As the acquisition of chaotic Raman scattering signals containing the temperature information within the FUT region gradually increases and decreases.

C-D segment is the stage when the pulse does not completely exit the FUT zone. This means that the Raman scattering signal intensity in the full pulse-width range is modulated by the temperature signal in the FUT. In theory, this part should exhibit a plateau because of the attenuation of the fiber, this indicates a downward trend.

E-F segment is the stage in which the pulse leaves the FUT completely and continues to propagate in the non-FUT zone. The transmission characteristics of chaotic signals in the fiber area provide a theoretical basis for chaos differential reconstruction technology.

### Chaos differential reconstruction and double correlation demodulation principle

**Fig. 14:**
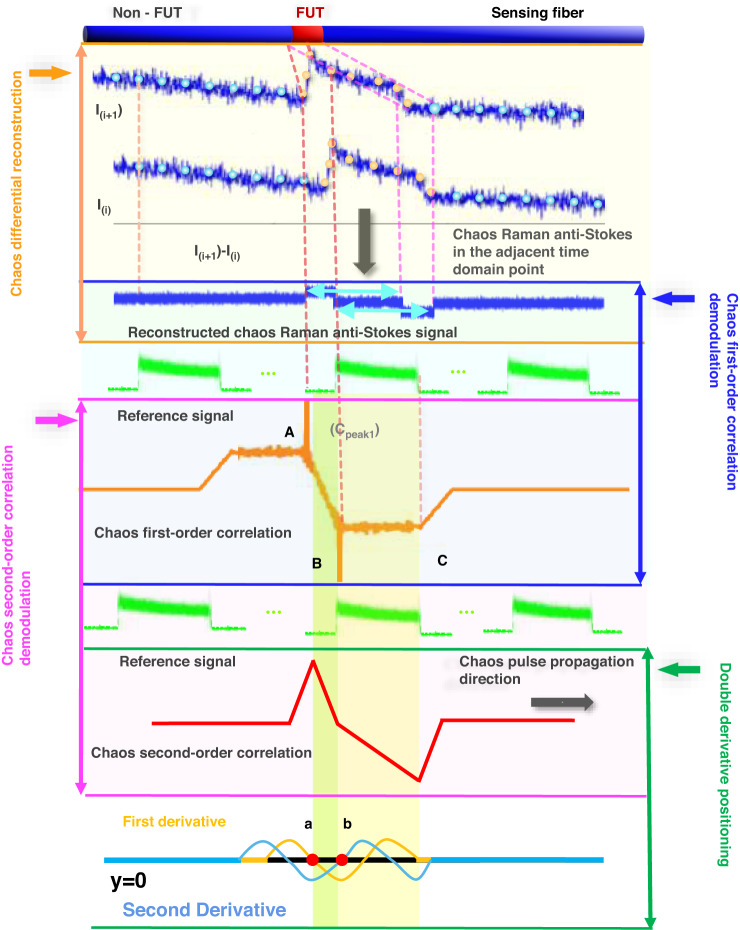
Principle of chaos differential reconstruction and double correlation demodulation in chaos Raman distributed optical fiber sensing scheme

According to the analysis of the generation characteristics of the above chaotic Raman scattering signals, each point collected by the system is the superposition of the backscattered signals over the pulse width. This process weakens the random fluctuation characteristics of chaos, making the chaos correlation characteristics unusable. Therefore, we need to extract the chaotic random fluctuation characteristics and temperature information of each point through chaos differential reconstruction processing. Simultaneously, this process can reduce the influence of the optical fiber attenuation coefficient on the experimental results. The chaos differential reconstruction principle is shown in Fig. 14 as the orange-colored area.

The functional purpose of chaos differential reconstruction method is to extract the chaotic Raman scattering signal with random fluctuation characteristics in the FUT region which is correlated with the chaotic reference signal. Then the temperature change information in FUT region from the Raman backscattering signal through the differential reconstruction operation of the adjacent position points in time sequence. The mathematical function equation of chaos differential reconstruction and the establishment process of the equation are shown as follows.

It can be seen from the Eq. (1) that the chaotic Raman anti-Stokes signal intensity *I*_*a*_(*L*) is proportional to *P*, *K*, *S*, *ϕ*_*e*_*, λ*_as_, and exp[−(α_0_ + α_as_) × *L*], and these physical quantities are not modulated by temperature. Therefore, these coefficients are unified into the constant *C*. The Eq. (3) is mathematical function equation of chaos differential reconstruction. After performing chaotic differential reconstruction on Eq. (1), the intensity of *I*_*c*_ after calculation can be expressed as:

3 $${I}_{c}(L)=C[P(n+1){R}_{as}({T}_{n+1})-P(n){R}_{as}({T}_{n})]$$

Then, we perform the chaos first-order correlation demodulation on the chaotic pulse signal and chaos differential reconstruction signal, after which we use the chaos correlation property to amplify the useful signal containing the temperature information and form the correlation peaks in a time sequence to detect the FUT along the sensing fiber. The corresponding principle is shown as the blue-colored region in Fig. 14. The physics principle of chaos first-order correlation demodulation is as follows.

In the chaos first-order correlation demodulation stage, the pulse width can be discretized into *m* data points, while the differential reconstruction signal can be discretized into *n* data points, which is determined by the fiber length and sampling rate of system. The total length of the sequence after first-order correlation calculation of these two signals is (*m* + *n* − 1), since the start position point (*m* − 1) and the end position point (*m* − 1) of the sequence are invalid data, the number of the valid data is (*n* − *m* + 1). The mathematical function equation of the chaos first-order correlation demodulation is shown in the Eq. (4). Among them, *P*_*r*_ is the reference signal, *I*_*c*_ is the signal intensity after chaotic differential reconstruction.

4 $${I}_{1}(i)=\mathop{\sum }\limits_{j=0}^{m-1}{P}_{r}(j){I}_{c}(i+j),i\in [0,n-m+1]$$

The chaos second-order correlation demodulation principles are shown in pink color in Fig. 14. Due to the influence of noise, the correlation peak (*C*_*peak1*_, position *A*) generated by the chaos first-order correlation demodulation method is not obvious, making the positive and negative correlation peaks unclear, and it leads to difficult to locate the regional position of FUT region. Then we use the reference signal and the signal after the chaos first-order correlation demodulation processing for chaos second-order correlation demodulation. In Fig. 14, the orange curvy is the signal after chaos first-order correlation demodulation and the green part is the chaotic pulse signal. The chaos second-order correlation demodulation signals are shown in the red curve.

The chaos second-order correlation demodulation can amplify the useful information (correlation peak of the chaos first-order correlation demodulation). It also can generate a pair of positive and negative chaos correlation peaks, wherein the positive peak corresponds to the positive peak of the original correlation signal, that is, to the starting position of the FUT zone. However, owing to the influence of the chaos pulse, the negative chaos peak position after chaos second-order correlation demodulation does not coincide with the negative peak position after chaos first-order correlation demodulation. Therefore, we finally use the derivative operation (first derivative and second derivative) to accurately locate the regional location information of FUT. The physics principle of the derivative operation is as follow.

In derivative calculation process, we use the first derivative to determine the starting point of the FUT region, and use the second derivative to calculate the end point of the FUT region. The specific positioning principle is as follows. In Fig. 14, the chaos second-order correlation demodulation signal shows an overall downward trend in the yellow and green-shaded areas (zones A-C). Among them, the corresponding decline rate of the signal after chaos second-order correlation demodulation in the green-shaded area (A-B zone) is relatively large. But in the B-C zone, its decline rate will become gentle compared with the signal in the A-B zone. Combined with correlation demodulation principle, the point A can as the coordinate origin. When the chaos pulse signal moves in the A-B zone under the correlation demodulation process, the intensity of the chaos second-order correlation (*I*_2_) can be expressed according to the definition of the chaos second-order correlation demodulation as the Eq. (5).

5 $${I}_{2}={C}_{peak2-}{\int }_{0}^{x}({C}_{peak1}-{f}_{X}(t))g(t)dt$$

Then we explain the Eq. (5) in detail. For convenience of the subsequent proof, we denote *I*_2_ as a continuous signals (compared to discrete signals). Among them, the *C*_*peak1*_ represents the correlation peak after chaos first-order correlation demodulation. *f*_*X*_(*t*) denotes the intensity after chaos first-order correlation demodulation. The positive and negative correlation peaks after chaos first-order correlation demodulation satisfy the Lorentz function, so the *f*_*A*_(*t*) can denote the intensity near the positive peak, which can be expressed as *f*_*A*_(t) = *C*_*peak1*_/(1 + *t*^2^), and the intensity near the negative peak is *f*_*B*_(*t*) =−*C*_*peak1*_/(1 + (*t* −*x*_*B*_)^2^)^2^, where *x*_*B*_ denotes the coordinate of point *B*. The *g*(*t*) denotes the intensity of the chaos signal. It is the same as the definition of *A*_*chaos*_ below.

Then we carried out the precise position of the FUT region by the characteristics of different falling slopes in the FUT region after the chaotic secondary correlation signal. The specific principle and demodulation process are as follows. The first-order and second-order derivatives of Eq. (5) are *I*_2_^′^ = −*A*_*chaso*_(*C*_*peak1*_ *−* *f*_*X*_(*x*)) and *I*_2_^″^ = *A*_*chaos*_*f*_*X*_^’^(*x*). Among them, when *x* = 0, *f*_x_(*x*) = *f*_*A*_(*x*) and *I*_2_^′^ = 0. When x > 0, *I*_2_^′^ < 0 indicates a decreasing trend. Therefore, *I*_*2*_ reaches its maximum value at point *A*. When *x* = *x*_*B*_, *f*_x_(*x*) = *f*_*B*_(*x*) and *I*_2_^″^ = −2*C*_*peak1*_*A*_*chaos*_(*x*-*x*_*B*_)/(1 + (*x*-*x*_*B*_)^2^) = 0. When *x* > *x*_*B*_, *I*_2_^″^ > 0; when *x* < *x*_*B*_, *I*_2_^″^ < 0. Therefore, *x* = *x*_B_ is the inflection point for *I*_2_. When 0 < *x* < *x*_*B*_, *f*_*X*_(*x*) ≈ −2*C*_*peak1*_ *x* + *C*_*peak1*_ and *I*_2_^″^ = 0.

Through the above analysis, the first-order derivative of *I*_2_ corresponds to the position of the positive peak after the chaos first-order correlation, that is, the starting position of the temperature change zone. The closest inflection point of *I*_2_ behind the starting position can be located at the negative peak position, that is, the end position of the temperature change region. In summary, the location and length of the temperature change zone can be determined, as indicated by points *a* and *b* in Fig. 14.

Further theoretical analysis, the chaotic pulse laser can be used as a set of pulse sequences composed of multiple narrow sub-pulses with random intensity fluctuations. All sub-pulses are effective for correlation demodulation regardless of their intensity, and they are also key parts of chaos differential reconstruction. This is because all the sub-pulse sequences will retain the time-series random fluctuation characteristics of the chaotic signal in the excited Raman scattering signal when passing through the FUT region. Therefore, when the temperature of the FUT region changes, the random fluctuation characteristics of these chaotic signals will also change. We can extract and locate these weak temperature changes by correlation demodulation with the reference signal.

On the contrary, if a limited number of sub-pulses with larger amplitude are used for differential reconstruction, the time series random fluctuation characteristics of the chaotic Raman scattering signal after differential reconstruction will become incomplete and inaccurate, which will lead to demodulating the wrong FUT region location and temperature information, and even unable to extract the temperature information.

### Physical characteristics of chaos correlation peaks and surrounding temperature

We demodulated the temperature change along the sensing fiber based on the chaos correlation peaks. The chaos differential reconstruction method based on Eq. (1) is performed, and because the attenuation coefficients of the two adjacent points are similar, they are approximately the same and are unified as a constant *C*. Therefore, the intensity after the difference operation Ic can be expressed as Ic = C [P(n + 1)Ras(Tn + 1)-P(n)Ras(Tn)]. *P* denotes the power of the input fiber. For the chaotic pulse signal, the number of data points corresponding to the chaos pulse width is m, whereas the number of data points for the differential signal is *n*, which is determined by the length of the fiber and the sampling rate. The total length of the sequence after the correlation operation of the two signals is (*m* + *n* − *1*), but the beginning (*m* − *1*) and end (*m* − *1*) data points of the sequence are invalid, and the valid data are (*n* *−* *m* + *1*). In the correlation calculation, the data when the chaos pulse is completely in the FUT zone are expressed as ${I}_{1}(i)={\sum }_{j=0}^{m-1}{P}_{r}(j){I}_{c}(i+j),i\in [0,n-m+1]$, where *P*_*r*_ is the chaos pulse signal power and *P*_r_:*P* = 1:99. Based on this, the above equation can be converted into

6 $$\begin{array}{l}{I}_{1}(i)=99\cdot C\cdot \mathop{\sum }\limits_{j=0}^{m-1}{P}_{r}(j)\left[\right.{P}_{r}(j+1)\cdot {R}_{as}({T}_{i+j+1})\\\qquad\quad -\,{P}_{r}(j){R}_{as}({T}_{i+j})\left.\right]i\in [0,n-m+1]\end{array}$$

The maximum correlation peak is generated when the FUT zone is in the chaotic pulse. Thus, the correlated peak-to-peak *C*_*peak1*_ can be expressed as

7 $$\begin{array}{l}{c}{C}_{peak1}=99\cdot C\cdot Ras({T}_{FUT})\mathop{\sum }\limits_{j=0}^{m-1}[{P}_{r}(j){P}_{r}(j+1)-{P}_{r}^{2}(j)]\\\qquad\qquad\,i\,\in [0,n-m+1]\end{array}$$

Then, the intensity *I*_2_ after the chaos second-order correlation can be expressed as ${I}_{2}(i)={\sum }_{j=0}^{m-1}{P}_{r}(j){I}_{1}(i+j),i\in [0,n-2m+2]$. Therefore, the peak-to-peak value of *C*_*peak2*_ after the chaos second-order correlation can be expressed as

8 $$\begin{array}{l}{C}_{peak2}=99\cdot C\cdot {R}_{as}({T}_{FUT})\mathop{\sum }\limits_{j=0}^{m-1}\mathop{\sum }\limits_{j=0}^{m-1}[{P}_{r}^{2}(j){P}_{r}(j+1)-{P}_{r}^{3}(j)]\\ \qquad\qquad i\in [0,n-2m+2]\end{array}$$

Because *P*_*r*_(*j*)_Max_I_1_ ≤ *P*_*r*_*(j*)*I*_*1*_ ≤ *P*_*r*_(*j*)_*Min*_*I*_*1*_, according to the Lagrange mean-value theorem, there must be a constant *P*_*c*_ such that *P*_*c*_*I*_*1*_ = *P*_*r*_(*j*)*I*_1_. To simplify the calculation, let *P*_*c*_ be approximately equal to ${\bar{P}}_{r}$, which is the average value of *P*_*r*_. Therefore, ${C}_{peak2}=m{\bar{P}}_{r}{C}_{peak1}$. Thus, the chaos second-order correlation amplifies the result after the chaos first-order correlation. The effects of some parameters and the loss of the fiber were not obvious in the experiment, but these parameters were present in the collected backscattered signal. Thus, we can demodulate the temperature information from the correlation peak using the backscattered signal after the chaos first-order correlation.

9 $$A=\frac{{C}_{peak2}}{{I}_{a}}=\frac{{R}_{as}({T}_{FUT})[{\sum }_{j=0}^{m-1}[{P}_{r}(j){P}_{r}(j+1)-{P}_{r}^{2}(j)]]}{{R}_{as}({T}_{0})}$$

By combining Eq. (2) with the above equation, the temperature demodulation formula can be obtained as shown in Eq. (10).

10 $${T}_{FUT}=\frac{h\varDelta v}{k\cdot {\rm{l}}n\left[1+\left(\tfrac{[{\sum }_{j=0}^{m-1}[{P}_{r}(j){P}_{r}(j+1)-P_{r}^{2}(j)]]}{A\cdot {[\exp (h\varDelta v/k{T}_{0})-1]}^{-1}}\right)\right]}$$

## Data Availability

Data underlying the results presented in this paper are not publicly available at this time but may be obtained from the authors upon reasonable request.
